# Dataset for the subnational seats of G20 countries subnational units

**DOI:** 10.1016/j.dib.2026.112851

**Published:** 2026-05-12

**Authors:** Nicolas Lucic

**Affiliations:** a Graduate School of Advanced Integrated Studies in Human Survivability (GSAIS), Kyoto University, Higashi-Ichijo-Kan, Yoshida-Nakaadachicho 1, Sakyo-ku, Kyoto 606-8306, Japan; b UMI Soutenabilité et Résilience (SOURCE), University of Versailles Saint Quentin-Paris Saclay, 47 Boulevard Vauban, 6F, Guyancourt 78280, France

**Keywords:** Representation, Legislatures, Subnational representatives, Elections, Political systems, Sublinear scaling, Population size

## Abstract

Assembly size has been a central question in the study and quest for proper political representation, going back thousands of years, at least back to Ancient Greece with discussions pertaining to the number of citizens per representative. Since then, political systems have evolved and formed rules to elect or appoint representatives. More recently, at least since Penrose (1946) and Taagepera (1972), social sciences have tried to understand the link between population growth and assembly size. At the national/federal levels, this subject is relatively adequately studied with hundreds of papers. Surprisingly enough, almost no research has tried to describe this relationship at the subnational level. A few have studied the political effects of assembly size at the micro level (municipal and district). This is probably due to the difficulty of gathering thousands of data points for subnational territories. This data paper aims at filling this gap, thus allowing for further systematic study of the link between assembly size, population size, population density, and number of subnational units.

To this end, data for all 19 sovereign countries of the G20 group were collected. This sample provides a compromise between data availability, political and economic diversity and world population coverage. Collection was done by cross-comparing national/state election reports with each subnational unit’s website. At total of 805 subnational units were identified, with a total of 69,110 subnational seats gathered, as of December 2025, constituting the main innovation of the present paper. The dataset has two forms, either an individual country perspective with 19 files or a single general file, compiling all data, sorted from smallest to biggest subnational unit population, thus pooling all countries. Australia, France, U.S.A., P.R. China and the United Kingdom have at least two sheets per file to differentiate between mainland only perspective or with overseas and special territories. For the general file compiling all data, there are also two sheets, one with island and special territories and the other with mainland only. This constitutes a minor innovation in the literature since very small and overseas territories (with <500 000 people) are never included in existing studies, thus potentially overlooking a different dynamic for the scaling relationship between population size and legislature size.

Researchers are encouraged to study the included country’s subnational units, in part or as a whole, as well as adding their own observations, as the dataset forms a basis for future expansion. One should note that as election results, and population size change quickly, one should understand the present paper as a late-2025 snapshot and look for an updated version in the future.

Specifications Table

**Table:** 

Subject	Social Sciences
Specific subject area	Political Science – Political Representation Subnational legislature size and sublinear relationship to population.
Type of data	Table, Textual, Numerical data. Raw and analyzed data. .xlsx numerical data .xlsx textual data
Data collection	Systematic exploration of subnational legislature websites, and, if available, of national election trackers’ websites. If none of those primary sources were available, then cross-checked media articles were used. No particular instrument needed. All available data within the scope were included, besides Russia and India. Reasons can be found in the READ_ME_Guide. All sources for individual data can also be found in the repository in the READ_ME_Sources.
Data source location	As data were collected online, there is no single location where data were collected. The data would match the observed number of seats in each of the 804 physically existing subnational units reported.All data are physically stored at GSAIS, Kyoto University, Higashi-Ichijo-Kan, Yoshida-Nakaadachicho 1, Sakyo-ku, Kyoto, 606–8306, Japan.Finally, subnational seats are formally secondary data but they strictly correspond to observable reality and are not transformed thus there is no physical source location. For population and land mass data, they are directly (with no transformation) taken from national statistical offices latest censuses/reports. For example, for France: - Institut National de la Statistique (INSEE), 88 Avenue Verdier, Montrouge, 92,000, France. One can follow this exact framework for every other country. All sources can be found in the READ_ME_Sources in the data repository.
Data accessibility	Repository name: Harvard Dataverse – G20 subnational legislatures Data identification number: [1] 10.7910/DVN/PEE4Z6 Direct URL to data: https://dataverse.harvard.edu/dataset.xhtml?persistentId=doi%3A10.7910%2FDVN%2FPEE4Z6 Instructions for accessing these data: Go to Harvard Dataverse, either look for Nicolas LUCIC’s Dataverse, or alternatively within the Harvard Dataverse, look for “G20 subnational legislatures”.
Related research article	None, future research will use this dataset.

## 1 Value of the Data

• This data paper is the first systematic exploration of the size of subnational legislatures, applied to G20 countries. It gathers all subnational seats, population and size in km² on a per subnational legislature basis. Its major innovation is the identification and inclusion of 805 subnational units amounting to 69 110 subnational seats.
• This data paper includes a secondary scientific contribution as it includes overseas and special territories (that have different relations between local government and population size) whereas literature has always focused on mainland territories only.
• Previous studies were limited to incomplete national samples or tended to focus on the same countries, restraining interpretability and potentially biasing all analyses trying to derive generalizable features.
• Researchers and public policy experts can use these data to study links between population, land mass, number of subnational units and number of subnational seats. One can also use these data as part of a larger research project focusing on the *effects* of the number of seats, for example on public spending, economic growth, trust and confidence in institutional levels and various other topics in economics or political science.
• This data paper will help future research in determining empirical and theoretical optimal political representation models insofar as it encompasses the largest number of institutional unit legislatures and allows for the study of politically and economically diverse territories.

## 2 Background

Is there an optimal size to a representative legislature? Since [2] Penrose (1946), [3] Taagepera (1972) and [4] Stigler (1976), this question has been hotly debated, and literature has largely concluded that the number of seats should sub-linearly scale with population, as recently confirmed by [5] Auriol and Gary-Bobo (2012). The value of sublinear scaling is now the center of attention.

Subnational legislatures have largely been an afterthought of research in this subsection of political science. Besides U.S. States, studied by [6] Sandoval (2023) and a literature review [7] (De Santo and Le Maux, 2023) that included some subnational legislatures, no other work has focused on the specific study of the relation of population to the size of subnational legislatures, outside of a previous attempt by [8] Tanimoto and Zhao (2023). This is due, in great part, to the lack of centralized data which hinders the research community's ability to work on the subject at hand. This data paper aims to bridge the gap.

Moreover, mainland territories have always been considered in the literature. Their small and/or overseas territories have never been included, which we correct to avoid skewing future interpretation.

## 3 Data Description

There are 19 country-specific files that will be described alphabetically. The only file pertaining to all countries will be described after all country-specific files. For replicability purposes, all files follow the same organizational logic.

**Table:** 

Subnational Units	lower house seats	upper house seats	total seats (lower + upper)	population (2020–2025 census)	Population per local seat	Area km²

This table displays the column structure that every single country-specific file follows. What differs between individual countries is the number of lines (corresponding to the number of subnational units considered) and the figures for each individual cell. Individual column explanation below:

*Subnationl Units*: lists individual names of subnational units, usually following alphabetical order (with this rule differing when overseas and special territories are considered). When the original language was understandable for an English-based reading audience, the name of the subnational unit was left in original language. If needed, subnational units names were transliterated to names commonly used internationally (for Saudi Arabia or P.R. China for example).

*Lower house seats*: displays the number of total seats (both filled and vacant) for subnational lower houses. In the common language, these can be named parliaments, chambers, legislatures, councils, assemblies or houses. For simplicity, all of these, even if they do not have the same local powers, professionalization, funding and political legitimacy, have been grouped together under the name *lower house*. Note that if a particular subnational unit is unicameral (as most are), seats were counted as lower house seats.

*Upper house seats*: displays the number of total seats (both filled and vacant) for subnational upper houses. Various names for upper houses were included. They are consistent as upper houses at the subnational level can usually be found in federal systems, which is verified here. Thus, most lines in this column remain blank.

*Total seats (lower + upper)*: total by adding lower and upper house seats for each individual subnational unit. Note that, by construction, an upper house and a lower house seat usually overlap per district. For example, in the U.S. system, the same elector can, at the same time, vote for a state representative and a state senator. Future research should take this into account.

*Population (2020–2025 census)*: displays the total population (resident + immigrant) per subnational unit as listed by the latest census. As citing each individual data source would be very tedious, especially for future researchers, when it was possible, we gathered data from a central source that would systematically list all subnational units’ population, after cross-checking their validity as reported by the national/local statistical office.

*Population per local seat:* displays the value of total seats divided by total population of each individual unit.

*Area km²*: displays the total land mass (including internal water area) of the country, including overseas territories when applicable.

The individual countries represented by individual files are presented below:

- *Argentina subnational legislatures 2025*: this file has one sheet with 24 subnational units with the total added as the last line.
*Australia subnational legislatures 2025*: this file has two sheets with first named *AUS with islands* having 10 subnational units (with islands added) and the second named *AUS without islands* having 8 subnational units, the total is added as the last line for both sheets.
- *Brazil subnational legislatures 2025*: this file has one sheet with 27 subnational units with the total added as the last line.
*Canada subnational legislatures 2025*: this file has one sheet with 13 subnational units with the total added as the last line.
- *France subnational legislatures 2025*: this file has three sheets with first named *FRA with all islands* (with all islands added), the second named *FRA regions with islands* having 18 subnational units, and the third sheet named *FRA mainland only* having 13 subnational units, with, for all three sheets, the total added as the last line.
*Germany subnational legislatures 2025*: this file has one sheet with 16 subnational units with the total added as the last line.
- *India subnational legislatures 2025*: this file has two sheets with first named *IND* having 32 subnational units (with Ladakh UT added) and the second named *IND without Ladakh UT* having 31 subnational units, the total is added as the last line for both sheets.
*Indonesia subnational legislatures 2025*: this file has one sheet with 38 subnational units with the total added as the last line.
- *Italy subnational legislatures 2025*: this file has one sheet with 20 subnational units with the total added as the last line.
*Japan subnational legislatures 2025*: this file has one sheet with 47 subnational units with the total added as the last line.
- *Mexico subnational legislatures 2025*: this file has one sheet with 32 subnational units with the total added as the last line.
- *P.R. China subnational legislatures 2025*: this file has two sheets with first named *P.R. China with SAR* having 33 subnational units (with Hong-Kong and Macau added) and the second named *P.R. China without SAR* having 31 subnational units, the total is added as the last line for both sheets.
- *Republic of Korea subnational legislatures 2025*: this file has one sheet with 32 subnational units with the total added as the last line.
- *Russia subnational legislatures 2025*: this file has two sheets with first named *RUS* having 85 subnational units and the second named *RUS without Crimea* having 83 subnational units, the total is added as the last line for both sheets.
- *Saudi Arabia subnational legislatures 2025*: this file has one sheet with 13 subnational units with the total added as the last line.
- *South Africa subnational legislatures 2025*: this file has one sheet with 9 subnational units with the total added as the last line.
- *Turkey subnational legislatures 2025*: this file has one sheet with 81 subnational units with the total added as the last line.
- *U.K. subnational legislatures 2025*: this file has six sheets; the first two represent the whole U.K. with and without the overseas territories, with the first named *GBR with BOT* (British Overseas Territories) having 228 subnational units, the second named *GBR without BOT* having 216 subnational units, the third sheet named *England* having 151 subnational units, the fourth sheet named *Scotland* having 32 subnational units, the fifth sheet named *Wales* having 22 subnational units, and the sixth sheet named *Northern Irland* having 11 subnational units. For all six sheets, the total was added as the last line.
- *USA subnational legislatures 2025*: this file has two sheets with first named *USA with overseas territories* having 56 subnational units (with islands added) and the second named *USA mainland only* having 50 subnational units (U.S. States), the total is added as the last line for both sheets.

## 4 Experimental Design, Materials and Methods

There is no particular structured methodology this data paper has followed, besides rigorous research and sourcing for all individual data pertaining to subnational institutional seats.

I have followed a simple and effective framework to corroborate and cross-check all data. First, if available, national elections board or formal reports were inspected. If so, these data were selected and cited. If not, the second phase was then deployed whereby I would visit individual subnational legislature websites to find, translate and locate the information (and usually manually count the number of legislators). Finally, when both of these methods proved unsuccessful, I turned towards secondary sources, mostly reputable national news providers, that sometimes have better and more up-to-date information than official sources, for example some subnational units in Indonesia, India, Russia and Turkey (mostly rural ones) do not clearly report their legislators, their number or whether there are vacant seats or not.

When all of these were verified and results reported, I surveyed any other secondary websites or repositories gathering all sources for one country on one webpage for ease of access. For some countries, one could find such a repository on Wikipedia. Given the reputation and the nature of that particular website, data reported on Wikipedia was systematically checked. As of November 2025, when Wikipedia is cited in the sources, it means that the data were exactly as reported by official reports. Wikipedia is thus not the primary source, just the secondary reporting entity.

This framework was applied repeatedly to every single country to find all seats for each subnational unit. All individual details can be found in the file named: *READ_ME_Guide_lucic_G20_subnational_legislatures*, available in the data repository.

## Limitations

First, there is potential for confusion around the term subnational unit. Here, we refer to the first institutional level below the national level. For example, states in the U.S. and Germany, prefectures in Japan, regions in France and Italy, provinces in Argentina and Brazil. It excludes districts and municipal level institutions. Note that the U.K. uses a distinct system that has been discussed prior.

Second, as elections and appointments happen and rules change, the number of seats evolves rather quickly. The reported data should be considered as a stable snapshot of the number of subnational seats at the end of December 2025. Any change after that would not be counted.

Third, as subnational legislatures are less known and studied, so is the availability of the data at hand, especially for countries where information is more tightly controlled, such as Russia and P.R. China. Thus, for these countries, there might be some slight differences in observations, especially since the legislators can be forced to resign or moved to another legislature.

## Ethics Statement

The author confirms he has read and follow the ethical requirements for publication in Data in Brief. This publication did not involve any human subjects, animal experiments nor data collection from social media.

## CRediT authorship contribution statement

**Nicolas Lucic:** Data curation.

## Data Availability

Harvard DataverseG20 subnational legislatures (Original data).
